# Prosthesis Survival, Complications, and Maintenance of Telescopic-Crown-Retained Removable Dental Prostheses and Root-Cap-Retained Overdentures: A Retrospective Cohort Study

**DOI:** 10.3390/dj14090545

**Published:** 2026-09-01

**Authors:** Anastasia Timoschenko, Lea Stoilov, Michael Marder, Helmut Stark, Norbert Enkling, Dominik Kraus, Milan Stoilov

**Affiliations:** 1 Department of Prosthodontics, Preclinical Education and Dental Materials Science, University Hospital Bonn, 53111 Bonn, Germany; anastasia.timoschenko@ukbonn.de (A.T.); lea.stoilov@ukbonn.de (L.S.); michael.marder@ukbonn.de (M.M.); helmut.stark@uni-bonn.de (H.S.); enkling@uni-bonn.de (N.E.); dominik.kraus@ukbonn.de (D.K.); 2 Department of Reconstructive Dentistry and Gerodontology, School of Dental Medicine, University of Bern, 3012 Bern, Switzerland

**Keywords:** removable partial denture, double crowns, overdentures, root caps

## Abstract

**Objectives:** To compare prosthesis survival, complication-free survival, and maintenance-free survival between telescopic-crown-retained dentures (TCDs) and root-cap-retained overdentures with ball attachments (RCDs), and to evaluate associated clinical factors. **Methods:** This retrospective single-center cohort study included 654 unique patients, each contributing one eligible prosthesis (406 TCDs and 248 RCDs). Mean follow-up was 4.15 years for TCD and 2.75 years for RCD. Time-to-event outcomes were analyzed using Kaplan–Meier methods and multivariable Cox regression. Adjusted analyses were restricted to prostheses supported by one to three abutments. **Results:** Unadjusted prosthesis survival was higher for TCD (*p* = 0.0001), whereas prosthesis type was not clearly associated with loss after adjustment (RCD vs. TCD: HR = 1.28; 95% CI: 0.75–2.19). Single-abutment support and, in sensitivity analysis, point-like support were associated with higher loss hazards. The higher adjusted complication hazard for RCD was attenuated in sensitivity analysis. RCDs were consistently associated with earlier maintenance (average HR = 1.75; 95% CI: 1.43–2.13), with exploratory analyses suggesting a greater difference during early follow-up and among single-abutment prostheses. **Conclusions:** The support situation appears more relevant to prosthesis loss than the retention concept alone. RCDs show earlier first documented maintenance requirements, whereas evidence for an independent difference in complication risk is model-dependent.

## 1. Introduction

In many industrialized countries, complete edentulism is steadily declining because of improved preventive strategies, increased awareness of oral hygiene, and advances in restorative and periodontal therapy [1,2]. As a result, more patients retain a greater number of natural teeth into older age, and the proportion of individuals receiving fixed dental prostheses continues to rise [2]. Nevertheless, the demand for removable partial dentures (RPDs) remains substantial. Among younger seniors in Germany, more than one third (37.4%) wear removable dental prostheses, with combined fixed–removable restorations being the predominant type [2]. Population-based data from other European countries show a comparable situation. In Switzerland, nearly half of individuals aged 75 to 84 years use RPDs, and this proportion increases to approximately 60% among those aged 85 years and older. These figures underline the continuing importance of RPDs in prosthetic rehabilitation across diverse clinical conditions and patterns of residual dentition [3].

Within this therapeutic landscape, removable partial dentures retained by double crowns—particularly telescopic-crown-retained dentures (TCDs)—have proven successful over recent decades [4,5,6]. Their increasing use, especially those fabricated from non-precious alloys, reflects both economic and clinical considerations in partially dentate patients [7,8]. Compared with clasp-retained partial dentures, telescopic systems offer superior esthetic outcomes, support a more favorable long-term prognosis for both abutment teeth and prostheses, reduce the risk of secondary caries, allow for uncomplicated modification if an abutment tooth is lost, and transmit masticatory forces predominantly along the axial direction of the supporting teeth [5,6,7,9].

In recent years, various materials have been investigated for the fabrication of inner and outer copings in double-crown-retained RPD, including non-precious metal alloys, zirconia ceramics, and high-performance polymers [10,11,12,13,14]. Experimental and clinical studies have generally reported promising outcomes across these materials [11,12,13,14,15]. Traditionally, these copings were fabricated from precious or non-precious metal alloys using casting techniques; however, these procedures were time-consuming, technique-sensitive, and, particularly in the case of precious alloys, associated with high manufacturing and material costs [13,14,16]. With the increasing use of CAD/CAM technologies, precision milling has become more frequently applied, allowing for the use of alternative materials and potentially improving manufacturing standardization and efficiency [13,14,16].

Non-precious metal alloys have become well established in many clinical settings because of their cost-effectiveness and favorable handling characteristics. However, robust long-term clinical data remain limited. Existing retrospective studies on RPD retained by non-precious alloy telescopic crowns indicate overall acceptable survival rates and manageable complication profiles [8,17,18].

Telescopic systems may also stabilize abutment teeth with reduced periodontal support and potentially decrease tooth mobility [19,20]. However, the rigid splinting effect inherent to telescopic crowns can also increase mechanical stresses, which may contribute to an elevated risk of root or crown fractures in structurally compromised teeth [6]. In clinical practice, fractured abutment teeth or damaged telescopic crowns may require other strategies to maintain prosthesis function. In such circumstances, root caps supported by ball attachments can serve as secondary abutments, providing a resilient means of retention when a telescopic abutment can no longer be preserved. Root-cap-retained overdentures (RCDs) may therefore represent a practical option in cases of severely reduced or unfavorably distributed residual dentition, limited remaining tooth structure, or disadvantageous crown-to-root ratios [21,22,23]. In this approach, the remaining teeth are shortened and fitted with precision attachments mounted on cast post-and-core restorations, thereby enhancing retention and allowing controlled prosthesis movement under functional loading [24]. Ball attachments represent a widely used attachment concept for root-retained overdentures; in the cohort reported by Berger et al. [25], spherical male parts with Dalbo Classic or Dalbo^®^-Plus matrices accounted for the majority of attachment systems used.

These two abutment concepts—telescopic crowns and root-cap-retained attachments—are biomechanically distinct. Telescopic systems create a rigid, splinting connection between abutment teeth and the prosthesis, resulting in predominantly axial load transfer [9]. Root-cap systems with ball attachments, by contrast, incorporate resilient attachments that allow limited movement and distribute shear forces away from structurally vulnerable roots [26]. Such differences imply not only divergent load-transfer mechanisms but also potentially distinct patterns of technical and biological complications [27,28].

Within the German statutory standard-care system, a removable denture retained by up to three telescopic abutments or, alternatively, by up to three root-cap abutments constitutes an established treatment pathway for arches with no more than three remaining teeth. These two retention concepts represent distinct standard-care categories, whereas a mixed design combining telescopic and root-cap abutments within the same arch is not defined as a regular standard-care option. Despite the routine use of both approaches, direct comparative evidence remains scarce, as most existing studies assess telescopic or root-cap-retained prostheses independently [7,8,17,25,29,30]. Consistent long-term data from cohorts treated within a unified prosthodontic concept are therefore limited. Since prosthesis survival, complication profiles, and maintenance burden are central determinants of long-term treatment success and patient-related outcomes, a direct comparison of these approaches remains clinically relevant.

The primary objective of this retrospective single-center study was to compare prosthesis survival, complication-free survival, and maintenance-free survival between telescopic-crown-retained and root-cap-retained removable dental prostheses delivered between 1 January 2014 and 31 March 2026. Secondary objectives were to assess the influence of abutment number, abutment configuration, denture design, jaw location, age, and sex on prosthetic outcomes. Although the number of root-cap-retained cases was comparatively smaller, evaluating both treatment concepts may inform prosthetic decision-making, particularly in situations involving structurally compromised abutment teeth.

## 2. Materials and Methods

The Ethics Committee of the Medical Faculty of the University of Bonn reviewed the project (Reference No. 056/22; 8 February 2022) and determined that formal ethical consultation was not required for this retrospective analysis of data obtained during routine clinical care. The Committee raised no professional or ethical objections. The study was conducted as a retrospective, non-interventional cohort investigation at the Department of Prosthodontics, Preclinical Education, and Dental Materials Science, University Hospital Bonn. The reporting of this retrospective cohort study followed the Strengthening the Reporting of Observational Studies in Epidemiology (STROBE) recommendations.

**Figure 1:**
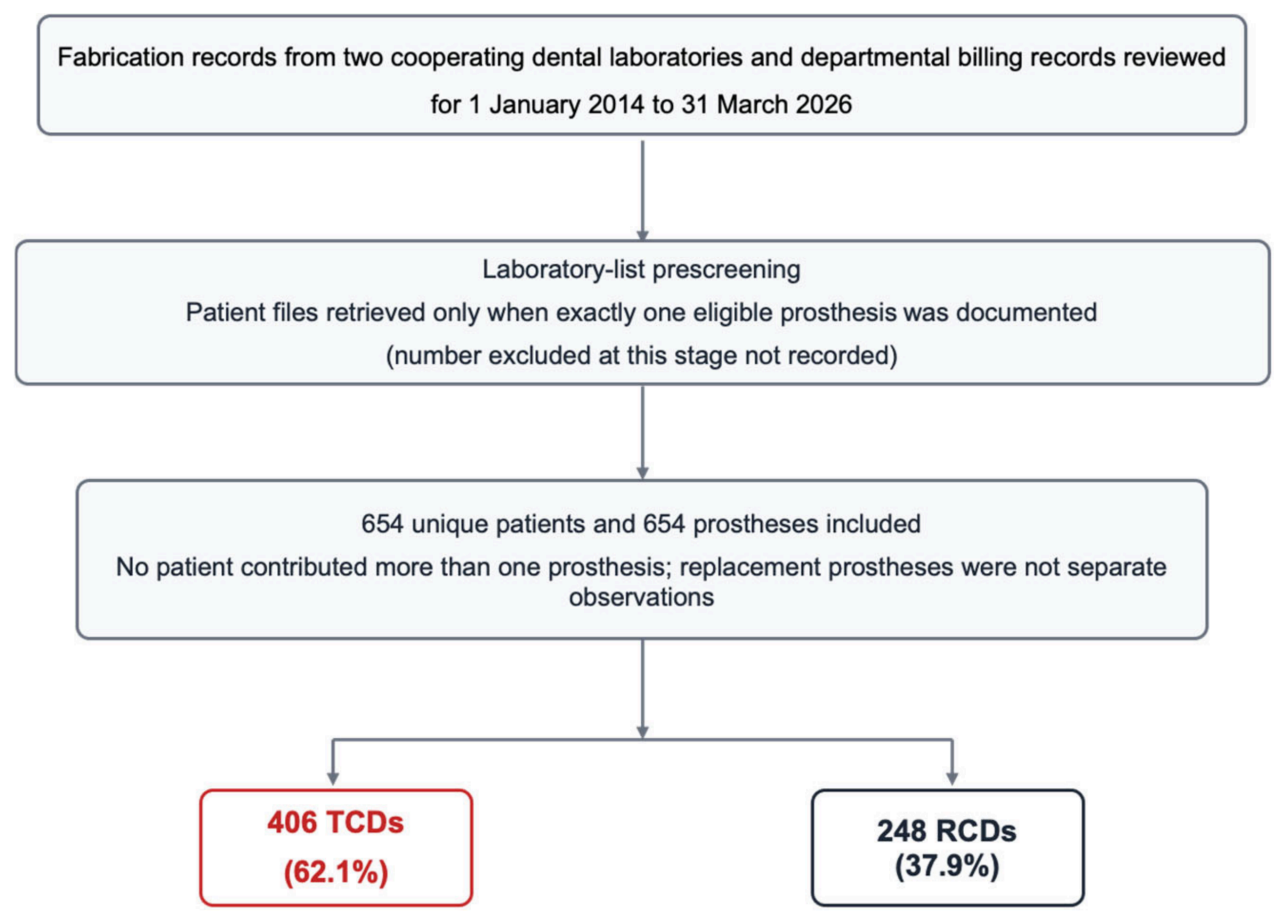
Flowchart of case identification and cohort assembly. Records from two cooperating dental laboratories and departmental billing data were reviewed from 1 January 2014 to 31 March 2026. The final cohort comprised 654 unique patients with 406 TCDs (62.1%) and 248 RCDs (37.9%); each patient contributed only one prosthesis.

Case identification was based on fabrication records provided by the two cooperating dental laboratories and on departmental billing records. These records were prescreened for prostheses delivered between 1 January 2014 and the data-lock date of 31 March 2026. Patient files were retrieved only when the laboratory records confirmed that exactly one eligible prosthesis had been fabricated for the patient. The final cohort therefore comprised 654 unique patients and 654 prostheses. No patient contributed more than one prosthesis, and replacement prostheses were not entered as separate observations. The number excluded during laboratory-list prescreening was not recorded separately and could not be reconstructed retrospectively (Figure 1).

Eligible prostheses were exclusively retained by telescopic crowns or by root caps with Dalbo Plus^®^ ball attachments (Cendres+Métaux SA, Bienne, Switzerland) and were fabricated from non-precious metal alloys. Prostheses incorporating additional retentive elements or implants were excluded. Paper records were reviewed manually and the study variables were extracted in pseudonymized form. The analysis dataset contained no missing values for the variables or outcomes evaluated. All eligible cases meeting these criteria were included; no a priori sample-size calculation was performed.

Patient variables were age at the time of prosthesis insertion and sex. Prosthesis variables comprised insertion date, last documented follow-up, jaw, prosthesis type, abutment number, Steffel support configuration [31], and denture-base design. An open design was defined as a prosthesis in which the denture base was relieved around the abutments, leaving gingival clearance to facilitate hygiene. In a closed design, the abutments or root caps were covered by a continuous denture base in the form of a cover denture or overdenture.

Prosthesis survival was defined as the time from insertion to complete prosthesis loss or remanufacture. Complication-free survival was defined as the time to the first documented prosthetic or technical complication, whereas maintenance-free survival was defined as the time to the first documented maintenance intervention. For each prosthesis, only the earliest documented complication date and the earliest documented maintenance date, respectively, were considered. If two or more event categories were documented on the same respective earliest date, they were treated as simultaneous first events. The prosthesis contributed a single event time to the corresponding time-to-event analysis, while all simultaneously documented categories were retained for the descriptive analyses presented. Events occurring after the respective first-event date were not included.

The complication endpoint was restricted to prosthetic and technical events. Data on biological events were not documented with sufficient completeness and standardization to permit their inclusion as a separate time-to-event endpoint. Data extraction and event classification were performed by two investigators, with each investigator’s assessments cross-checked by the other. Disagreements were referred to a third experienced investigator and resolved by consensus. No separate formal adjudication protocol was used; ambiguous cases were jointly reviewed according to the predefined event definitions.

### 2.1. Statistical Analysis

Statistical analyses were performed using GraphPad Prism^®^ 10, version 10.6.1 (799) (GraphPad Software, San Diego, CA, USA) and IBM^®^ SPSS^®^ Statistics, Version 31 (IBM Corp., Armonk, NY, USA). Continuous variables are presented as means with standard deviations or medians with ranges, as appropriate. Categorical variables are reported as absolute and relative frequencies.

Time-to-event analyses were conducted for prosthesis survival, first complication, and first maintenance. Prostheses without the respective event were censored at the last documented follow-up. For complication-free and maintenance-free survival, complete prosthesis loss or remanufacture occurring before the respective first event was treated as censoring at the date of prosthesis loss. Accordingly, the corresponding Cox regression models estimated cause-specific hazards among prostheses that remained in use and were free of the respective event. Adjusted hazard ratios (HRs) with profile-likelihood 95% confidence intervals (CIs) were reported. Tied event times were handled using Efron’s approximation in all Cox proportional hazards regression analyses. The proportional-hazards assumption was assessed graphically for each model coefficient using scaled Schoenfeld residuals plotted against follow-up time. Residual patterns were inspected for systematic time-dependent variation. Because GraphPad Prism^®^ did not provide a formal global Schoenfeld-residual test, the overall assessment of each model was based on the complete set of covariate-specific residual patterns. Where graphical time dependence was observed, Cox hazard ratios were interpreted as average associations over the observed follow-up period rather than as constant effects at every time point.

Unadjusted time-to-event distributions were estimated using the Kaplan–Meier method in the complete cohort of 654 restorations. Survival curves were compared using the log-rank (Mantel–Cox) test and the Gehan–Breslow–Wilcoxon test. Median event-free survival times were reported when estimable. Log-rank tests for trend were used considered for groups with an objectively ordered structure, such as increasing abutment number. Analyses involving small subgroups or no observed events were interpreted descriptively.

The primary objective of this study was to compare RCD and TCD with respect to time to first maintenance, first complication, and prosthesis loss. The associations of prosthesis support and denture base design with these outcomes were examined as exploratory, hypothesis-generating analyses during the analytical process.

Primary analyses comprised unadjusted Kaplan–Meier estimates with log-rank comparisons and multivariable Cox regression models adjusted for clinically relevant covariates available from the retrospectively retrieved clinical records (Covariates: age, sex, jaw location, and abutment number/Steffel-configuration). Although the addition of a quadratic age term slightly improved model fit for some of the models, the estimated hazard ratios for the groups of interest remained essentially unchanged across all outcomes. As the more complex specification did not alter the substantive conclusions, age was retained as a linear predictor in the final models to facilitate interpretation and preserve model parsimony.

Because RCDs were exclusively supported by one to three abutments, whereas the TCD group also included small numbers of restorations supported by four or more abutments, the adjusted multivariable Cox regression analyses were restricted to restorations supported by one to three abutments. This restriction defined the overlapping range of abutment support between both prosthesis types and reduced extrapolation beyond their common clinical support configurations. The resulting Cox regression cohort comprised 585 restorations.

Steffel-based sensitivity analyses were performed using an alternative representation of the support configuration [31]. In these sensitivity models, Steffel variable replaced categorical abutment number.

For the exploratory models including interaction terms, prosthesis support was dichotomized as one abutment versus two to three abutments (Steffel-based sensitivity analyses: point-like support and non-point-like support) to reduce model complexity and improve the stability of the parameter estimates. The interaction models included prosthesis type, dichotomized prosthesis support, their interaction term, age, sex, and jaw location.

Denture base design (open or closed) was closely associated with prosthesis type in the present cohort, with open designs predominantly used for TCD and closed designs for RCD. This distribution reflects the distinct clinical design principles of the two prosthetic concepts, which are not freely interchangeable. Denture design was therefore not included as an adjustment variable in the primary comparison between RCD and TCD. Given the limited overlap between design categories, such adjustment would have relied heavily on uncommon prosthesis type–design combinations and would have shifted the interpretation from the clinically relevant overall association between prosthesis type and outcome to an association conditional on denture design. Instead, an exploratory interaction analysis was performed to assess whether the association between prosthesis type and prosthesis survival differed according to denture base design. Because of the limited common support and small numbers in some combinations, these findings should be interpreted cautiously as hypothesis-generating.

All tests were two-sided, and no adjustment for multiplicity was applied. A *p*-value <0.05 was considered statistically significant. Interpretation also considered effect estimates, 95% confidence intervals, consistency across analyses, and clinical plausibility.

### 2.2. Fabrication and Insertion

Treatment was provided within the departmental routine-care pathway by staff dentists and senior clinicians. Laboratory procedures were performed by two cooperating dental laboratories. Exact numbers of individual operators and technicians were not recorded in analyzable form. The general clinical and laboratory workflow was standardized, although operator, laboratory, and protocol variation over the 12-year period could not be modeled retrospectively. Conservative, periodontal, and functional pretreatment and comprehensive prosthetic planning preceded definitive treatment. In all cases, patient treatment was initiated only after comprehensive patient information had been provided and written informed consent had been obtained.

After provisional jaw relation recording, abutment teeth were prepared. Whenever possible, the abutment teeth were prepared with a common path of insertion using chamfer diamond burs with coarse (135 µm) and fine grit (40–50 µm).

Depending on the clinical situation, the finish line was placed epi- to subgingivally within sound dental hard tissue.

For abutments intended for root-cap-retained prostheses with ball attachments, the clinical crown was additionally reduced to a residual stump height of approximately two millimeters. The clinical root surface was required to be free of residual composite material. Post space preparation was performed using drills adapted to the root canal anatomy (ParaPost X; Coltène©, Altstätten, Switzerland). To preserve an adequate apical seal of the existing root filling, post preparation was limited to approximately four millimeters short of the apex. To reduce the risk of fracture of the cast post of the root cap, a canal inlay preparation was performed in the coronal portion of the root canal.

The prepared abutment teeth for telescopic primary crowns were impressed using a conventional two-step wash impression technique with high- and low-viscosity addition-curing silicone materials (Honigum Putty Rigid Fast and Honigum Light; DMG, Hamburg, Germany). The impression procedure for abutments prepared for root caps was performed using a double-mix technique. For this purpose, the prepared stump and an impression post were coated with low-viscosity silicone, and additional material was introduced into the post space. Subsequently, a partial impression was made with high-viscosity silicone (Honigum Putty Rigid; DMG, Hamburg, Germany) using inlay impression trays (Speiko, Bielefeld, Germany). 

Telescopic primary crowns (inner copings) were fabricated from a non-precious cobalt–chromium–molybdenum alloy using a CAD/CAM workflow (Zirkonzahn^®^, Gais, Italy), with an intended parallel design and a convergence angle of 0° between the axial walls of the inner copings and the corresponding outer coping. Root caps were fabricated in the dental laboratory using the lost-wax technique after wax pattern modeling. The intraoral fit of the inner copings and root caps was assessed using a low-viscosity silicone material (Xantopren L blue, Kulzer©, Hanau, Germany). In addition, marginal adaptation was evaluated clinically with a dental probe, and a defined, rotation-free fit was verified. To accurately transfer the intraoral position of the inner copings and the root caps, a pick-up impression was made using an individual tray and a polyether impression material (3M™ Impregum™ Penta™ Soft, Solventum©, Kamen, Germany). Vertical maxillomandibular relations were determined conventionally using wax rims. For all prostheses, the horizontal maxillomandibular relationship was recorded in centric relation using a CRS Set 15 gothic arch tracing system (needle-point tracing; Candulor©, Glattpark, Switzerland). The casts were subsequently mounted in an Artex CR articulator (Amann Girrbach©, Pforzheim, Germany) using the centric relation record and a facebow transfer.

**Figure 2:**
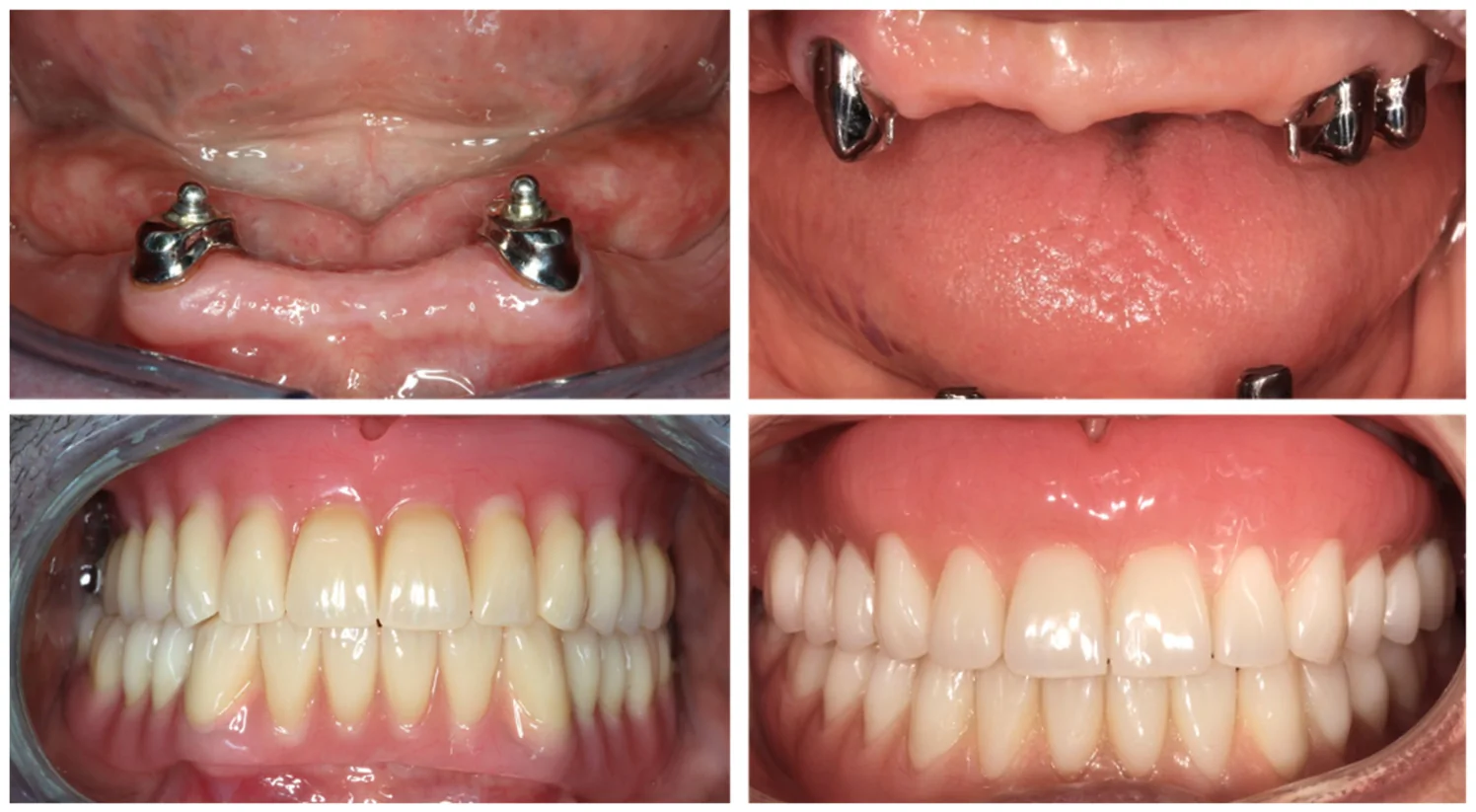
Clinical examples of removable partial dentures with different retentive elements and a closed cover-denture design. Left panels: mandibular root-cap-retained overdenture supported by two preserved roots with ball attachments. Right panels: maxillary telescopic denture retained by three telescopic crowns. Although the prostheses may resemble complete dentures when inserted, they are retained by preserved natural teeth or roots, as shown in the corresponding intraoral views. Written consent for publication of the photographs was obtained.

The secondary framework was likewise fabricated in the dental laboratory based on digital design data. During clinical try-in, the fit between the primary and secondary components and the intraoral fit of the secondary framework were assessed visually and tactilely. Stable, non-mobile seating of the prosthetic reconstruction was verified by axial loading of the secondary framework. The intaglio surface of the denture base as well as the adaptation of the transverse and/or sublingual bar were also assessed using low-viscosity silicone (Xantopren L blue, Kulzer©, Hanau, Germany). Tooth arrangement was performed in the dental laboratory within the neutral zone and adapted to the existing opposing dentition. The primary occlusal criterion was a stable static occlusion with evenly distributed bilateral contacts. Depending on the clinical situation and the number of remaining abutments, the dynamic occlusal scheme was established as bilaterally balanced, unilaterally balanced, or canine-guided, as appropriate. Tooth arrangement and occlusion were clinically verified before final processing of the prosthesis. Selection of an open or closed prosthesis design was based on the number, distribution, and strategic value of the remaining abutment teeth (Figure 2).

Before cementation, the inner copings and root caps were airborne-particle abraded (APA) in the dental laboratory using 50 µm aluminum oxide particles at 1 bar pressure. Definitive cementation was performed according to the location of the preparation margin and the ability to achieve adequate moisture control, using either a self-adhesive resin cement (3M™ RelyX™ Unicem 2, Solventum©, Kamen, Germany), a conventional resin cement (PermaCem; DMG, Hamburg, Germany), zinc phosphate cement (Hoffmann Dental, Berlin, Germany), or glass ionomer cement (3M™ Ketac™ Cem Plus, Solventum©, Kamen, Germany). For epigingival finish lines and adequate moisture control, 3M RelyX Unicem 2 or PermaCem was preferred, whereas for subgingival finish lines or compromised moisture control, zinc phosphate cement or glass ionomer cement was used preferentially. To ensure marginal integrity and a low-stress fit, the inner copings were definitively cemented first, followed by insertion of the secondary framework. In root-cap-retained overdentures, the matrix component was polymerized chairside into the secondary framework using a luting material (AGC Cem, C. HAFNER GmbH + Co. KG, Wimsheim, Germany) to achieve a precise fit between patrix and matrix.

After cementation, standardized patient instruction was provided, and insertion and removal of the prosthesis were practiced under supervision. In addition, all patients received standardized oral and prosthesis hygiene instructions.

Post-insertion control visits were generally arranged approximately one week after prosthesis delivery, although the exact timing was not standardized prospectively and depended on clinical need and patient attendance. No intentionally different follow-up schedule was applied to TCD and RCD. All documented interventions performed after the day of prosthesis insertion, including procedures during early adaptation visits, were classified as maintenance events. Adjustments performed on the day of insertion were not counted as maintenance interventions.

## 3. Results

### 3.1. Cohort Characteristics

A total of 654 prostheses were analyzed, comprising 406 telescopic-crown-retained removable dentures (TCDs; 62.1%) and 248 root-cap-retained overdentures (RCDs; 37.9%). Demographic and prosthesis-related characteristics are summarized in Table 1. Compared with the TCD group, the RCD group had a higher mean age and a shorter follow-up period. RCDs were predominantly located in the mandible, generally exhibited a closed design, and were more often supported by point-like configurations and fewer abutments. In contrast, TCDs were mainly open-design restorations with linear support configurations.

During the follow-up period, complete prosthesis loss occurred in 45 TCDs (11.1%) and 38 RCDs (15.3%), corresponding to 83 losses overall (12.7%). At least one complication was documented in 78 TCDs (19.2%) and 47 RCDs (19.0%), resulting in an overall complication rate of 19.1%. At least one maintenance intervention was recorded for 314 TCDs (77.3%) and 231 RCDs (93.1%), corresponding to an overall maintenance rate of 83.3%. The distributions of complication categories and maintenance interventions are presented in Table 2 and Table 3, respectively.

### 3.2. Prosthesis Survival

**Figure 3:**
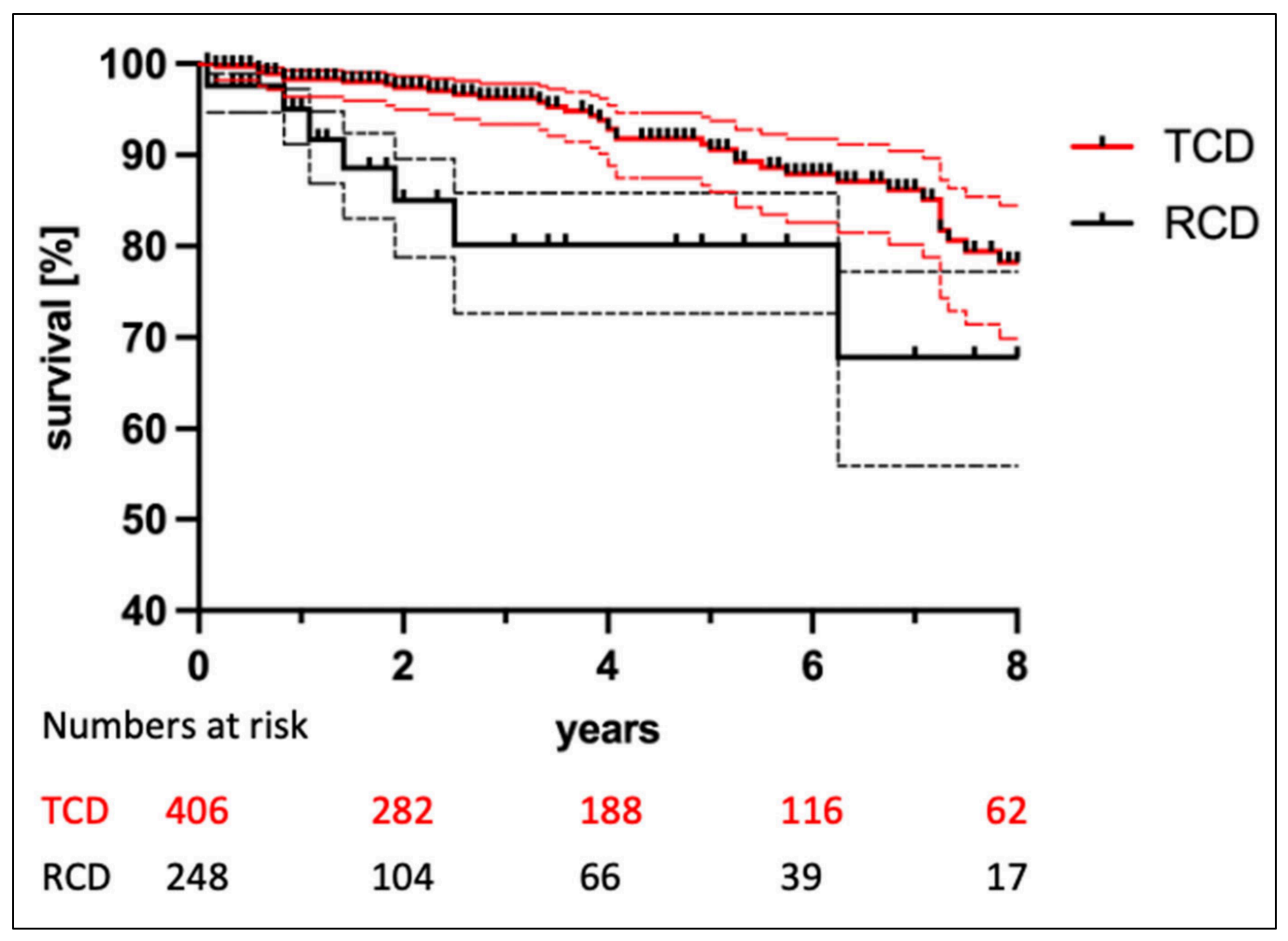
Kaplan–Meier estimates of prosthesis survival for TCD and RCD with pointwise 95% confidence intervals calculated using the log-log transformation and numbers at risk. Curves are truncated at 8 years because few RCDs remained at risk thereafter. Tick marks indicate censored observations.

Kaplan–Meier analysis of the complete cohort showed a separation of the unadjusted prosthesis-survival curves for telescopic-crown-retained dentures (TCDs) and root-cap-retained overdentures (RCDs) (Figure 3). The survival curves differed significantly in both the log-rank test (χ^2^ = 15.03, df = 1, *p* = 0.0001) and the Gehan–Breslow–Wilcoxon test (χ^2^ = 24.68, df = 1, *p* < 0.0001). The estimated 5-year prosthesis survival was 90.6% (95% CI: 85.9–93.7%) for TCD and 80.2% (95% CI: 72.6–85.8%) for RCD. Median prosthesis survival was not reached in either group during the follow-up period.

The adjusted analysis of prosthesis survival was performed in the restricted cohort of restorations supported by one to three abutments, representing the overlapping range of abutment support between both prosthesis types. The analysis included 585 restorations and 81 prosthesis-loss events. After adjustment for abutment number, age, sex, and jaw location, prosthesis type was no longer independently associated with prosthesis loss (RCD vs. TCD: hazard ratio [HR] = 1.28; 95% confidence interval [CI]: 0.75–2.19; Table 4). In contrast, restorations supported by a single abutment showed a significantly higher hazard of prosthesis loss than those supported by two abutments (HR = 3.67; 95% CI: 2.20–6.15). No significant difference was observed between restorations supported by three and two abutments (HR = 0.52; 95% CI: 0.23–1.06). The log-linear model additionally suggested a higher prosthesis-loss hazard with increasing age (HR per year = 1.05; 95% CI: 1.03–1.08). However, because the quadratic-age model provided a better fit (likelihood-ratio test: *p* = 0.0038; AIC: 799 vs. 805), this association is likely to have a stronger effect on elderly patients and should not be interpreted as a constant proportional increase per year.

The observed association between prosthesis support and prosthesis loss was confirmed in the Steffel-based sensitivity analysis, in which support configuration (point-like vs. non-point-like) replaced dichotomized abutment number in the adjusted Cox model. Prosthesis type remained unassociated with prosthesis loss (RCD vs. TCD: HR = 1.54; 95% CI: 0.92–2.59). Likewise, point-like support was associated with a significantly increased hazard of prosthesis loss compared with non-point-like support (HR = 2.88; 95% CI: 1.77–4.67). The corresponding unadjusted Kaplan–Meier curves stratified by prosthesis type and Steffel support configuration are shown in Figure S4.

In a further hypothesis-generating approach, we investigated whether the association between abutment support and prosthesis loss differed according to prosthesis type. Abutment support was dichotomized as one versus two to three abutments, and a prosthesis type-by-abutment support interaction term was included in the adjusted Cox model. No evidence of effect modification by prosthesis type was observed, as the interaction was not statistically significant (interaction HR = 1.27; 95% CI: 0.48–3.66; Table 5). For both prosthesis types, single-abutment support remained associated with an increased hazard of prosthesis loss compared with two to three abutments (HR TCD = 3.84; 95% CI: 1.83–7.51; HR RCD = 4.69; 95% CI: 2.24–9.80).

To further characterize the observed association with abutment support, exploratory unadjusted Kaplan–Meier analyses were performed according to abutment number across the full study population. Among TCDs, prosthesis survival differed across abutment-number categories (Figure S2; log-rank χ^2^ = 20.43, df = 3, *p* = 0.0001; Gehan–Breslow–Wilcoxon χ^2^ = 21.48, df = 3, *p* < 0.0001). Median survival was 7.08 years for single-abutment TCDs and 10.33 years for TCDs supported by two to three abutments, whereas median survival was not reached among TCDs supported by four or more abutments. No prosthesis losses occurred in the subgroup supported by more than five abutments.

A similar pattern was observed among RCDs (Figure S3), with significant differences in prosthesis survival across abutment-number categories (log-rank χ^2^ = 21.07, df = 2, *p* < 0.0001; Gehan–Breslow–Wilcoxon χ^2^ = 15.83, df = 2, *p* = 0.0004). Median survival was 6.25 years for single-abutment RCDs and was not reached for RCDs supported by two or three abutments. These findings provide an unadjusted characterization of the association between abutment number and prosthesis survival and complement the adjusted analyses described above.

### 3.3. Complication-Free Survival

**Figure 4:**
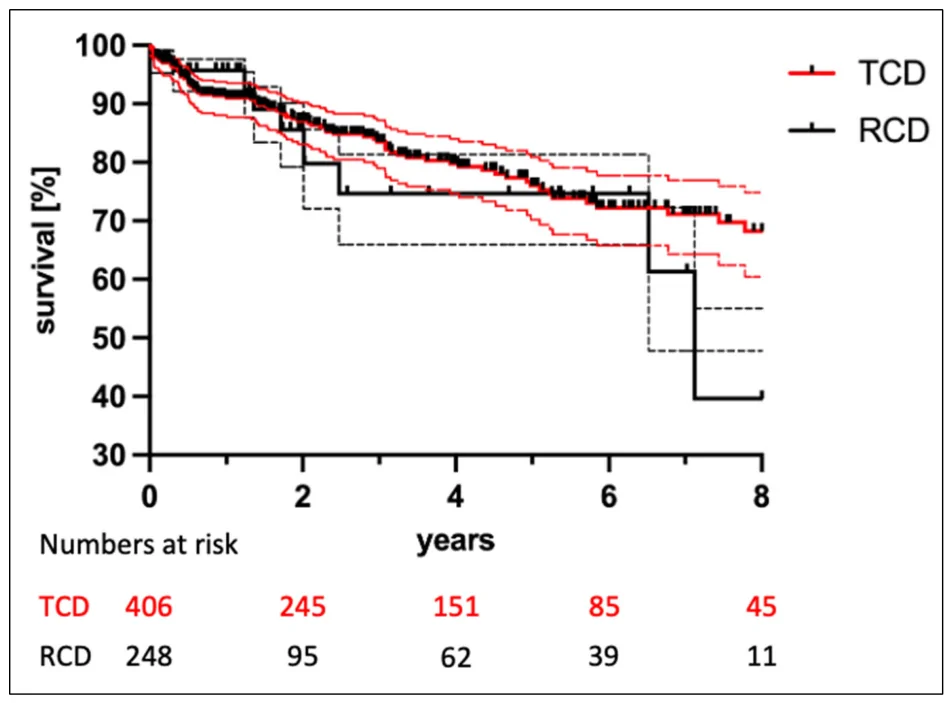
Kaplan–Meier estimates of complication-free survival for TCDs and RCDs with pointwise 95% confidence intervals calculated using the log-log transformation and numbers at risk. Curves are truncated at 8 years because few RCDs remained at risk thereafter. Tick marks indicate censored observations.

Kaplan–Meier analysis of the complete cohort showed broadly similar unadjusted complication-free survival curves for TCDs and RCDs (Figure 4). The log-rank test did not provide clear evidence of a difference between the groups (χ^2^ = 2.49, df = 1, *p* = 0.1143). The estimated 5-year complication-free survival was 76.0% (95% CI: 70.2–80.9%) for TCDs and 74.6% (95% CI: 66.0–81.4%) for RCDs. Median complication-free survival was not reached in the TCD group but was 7.12 years in the RCD group.

The adjusted multivariable Cox regression analysis included 585 restorations supported by one to three abutments, among which 108 first complications occurred. After adjustment for abutment number, age, sex, and jaw location, RCDs showed a significantly higher hazard of first complication than TCDs (HR = 1.81; 95% CI: 1.16–2.80; Table 6). Male sex was associated with a lower complication hazard than female sex (HR = 0.55; 95% CI: 0.37–0.82), and maxillary restorations showed a lower complication hazard than mandibular restorations (HR = 0.48; 95% CI: 0.31–0.73). Age and abutment number were not independently associated with complication-free survival.

In the Steffel-based sensitivity analysis, the estimated association between prosthesis type and complication-free survival remained directionally consistent but was attenuated and no longer statistically significant (RCD vs. TCD: HR = 1.51; 95% CI: 0.98–2.30). Consistent with the findings for abutment number, no independent association between support configuration and time to first complication was observed (point-like vs. non-point-like support: HR = 0.94; 95% CI: 0.60–1.44; Figure S5).

Exploratory analysis did not reveal a statistically significant interaction between prosthesis type and abutment support (interaction HR = 1.14; 95% CI: 0.40–3.79; Table 7). Point-like support was associated with a lower hazard in both prosthesis types, but this association was not significant. RCDs exhibited a higher hazard of first complication when compared with TCDs in both prostheses supported by one abutment (HR = 1.80; 95% CI: 0.66–5.07) and those supported by two to three abutments (HR = 1.62; 95% CI: 1.02–2.53) in the adjusted analysis.

### 3.4. Maintenance-Free Survival

Graphical Schoenfeld-residual diagnostics for maintenance-free survival suggested that the association between prosthesis type and the outcome was more pronounced during early follow-up. This pattern indicates that differences in hazard may arise predominantly during the early post-delivery period. Therefore, the Cox regression estimates reported below should be interpreted as average associations over the observed follow-up period rather than as constant effects at all time points.

**Figure 5:**
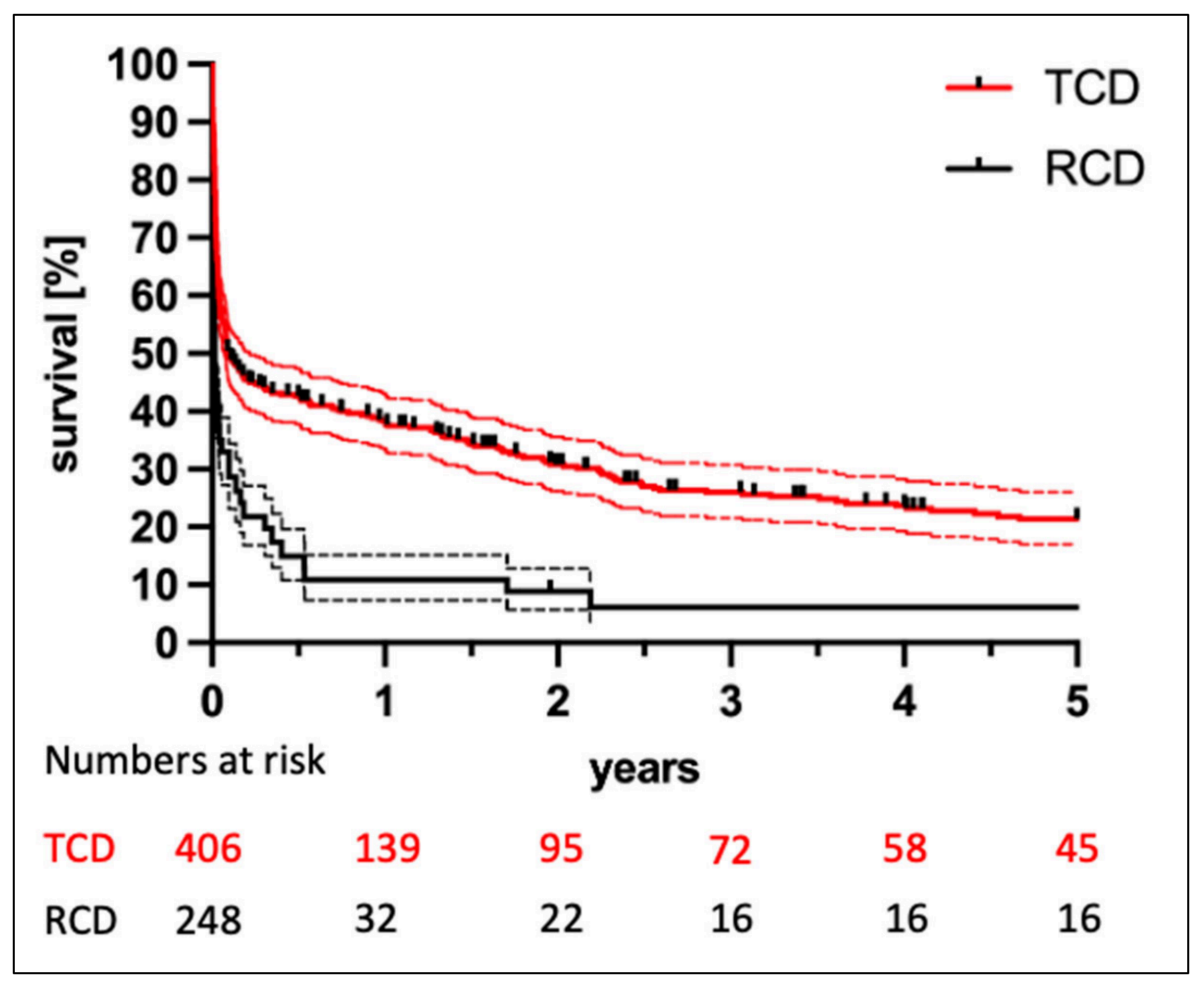
Kaplan–Meier estimates of maintenance-free survival for TCD and RCD with pointwise 95% confidence intervals calculated using the log-log transformation and numbers at risk. Curves are truncated at 5 years because few RCDs remained at risk thereafter. Tick marks indicate censored observations.

Kaplan–Meier analysis of the complete cohort showed a pronounced separation of the unadjusted maintenance-free survival curves for TCDs and RCDs (Figure 5). The log-rank test provided strong evidence of a difference between the groups (χ^2^ = 64.60, df = 1, *p* < 0.0001). The estimated 5-year maintenance-free survival was 21.3% (95% CI: 17.0–26.0%) for TCDs and 6.1% (95% CI: 3.5–9.8%) for RCDs. Median maintenance-free survival was 0.096 years in the TCD group and 0.016 years in the RCD group, corresponding to approximately 35 and 6 days, respectively.

The observed association remained largely unchanged after adjustment. The multivariable Cox regression analysis included 585 restorations supported by one to three abutments and 494 first-maintenance events. After adjustment for abutment number, age, sex, and jaw location, the Cox model yielded an average hazard ratio of 1.75 (95% CI: 1.43–2.13) for first maintenance comparing RCDs with TCDs (Table 8). This estimate was similar to the unadjusted analysis (HR_unadjusted_ = 1.91; 95% CI: 1.60–2.28).

In addition to prosthesis type, abutment support was also significantly associated with first maintenance. Compared with single-abutment restorations, both two-abutment (HR = 0.67; 95% CI: 0.53–0.84) and three-abutment restorations (HR = 0.83; 95% CI: 0.64–1.09) showed a lower estimated maintenance hazard; however, only the comparison between two- and single-abutment restorations reached statistical significance. The estimate for three versus two abutments suggested a small increase in maintenance hazard, but the confidence interval was very close to the null (HR = 1.253; 95% CI: 1.002–1.563).

In the linear age model, increasing age was associated with a higher hazard of first maintenance (HR per year = 1.012; 95% CI: 1.005–1.020). However, the addition of a quadratic age term improved model fit, indicating a non-linear relationship, with the increase in maintenance hazard leveling off at higher ages.

In the Steffel-based sensitivity analysis, the association between prosthesis type and maintenance-free survival remained unchanged (RCD vs. TCD: HR = 1.78; 95% CI: 1.46–2.15). The estimated effects of the Steffel support categories were directionally consistent with those observed for abutment number, although the estimates were less precise. Compared with point-like support, the hazard ratio for linear support was 0.88 (95% CI: 0.71–1.08) and 0.64 (95% CI: 0.42–1.00) for trilinear support.

Exploratory Analysis of maintenance-free survival revealed a significant interaction between prosthesis type and abutment support (interaction HR = 1.91; 95% CI: 1.20–3.11; Table 9). TCDs had a lower maintenance hazard than RCDs in both abutment-support groups, with the difference being more pronounced among single-abutment-supported restorations (HR = 2.65; 95% CI: 1.73–4.05) than among restorations supported by two to three abutments (HR = 1.45; 95% CI: 1.15–1.81).

Conversely, only within the RCD group, single-abutment support was associated with a higher maintenance hazard compared with two to three abutments (HR = 1.75; 95% CI: 1.34–2.30), whereas no clear association was observed within the TCD group (HR = 0.94; 95% CI: 0.62–1.37).

### 3.5. Exploratory Kaplan–Meier Subgroup Analyses

To retain the clinically informative unadjusted subgroup comparisons, additional Kaplan–Meier analyses were performed according to abutment number, Steffel support configuration, and denture design. These analyses complement the adjusted Cox regression models summarized in Table 4, Table 5, Table 6 and Table 7. The corresponding Kaplan–Meier curves are provided in Figures S1–S6.

#### 3.5.1. Prosthesis Survival According to Abutment Number

In an exploratory analysis restricted to restorations supported by one to three abutment teeth, the unadjusted prosthesis-survival curves showed separation between TCDs and RCDs (Figure S1). The log-rank test yielded χ^2^ = 9.58 (df = 1, *p* = 0.0020), and the Gehan–Breslow–Wilcoxon test was also significant (χ^2^ = 17.32, *p* < 0.0001). Median survival was 10.33 years for TCDs and was not reached for RCDs. These unadjusted findings should be interpreted alongside the adjusted analyses presented in Table 4 and Table 5.

In an exploratory within-group analysis, the unadjusted prosthesis-survival curves for TCDs showed heterogeneity across categories of abutment number (Figure S2). The global log-rank test provided evidence of differences among the curves (χ^2^ = 20.43, df = 3, *p* = 0.0001), with similar results obtained using the Gehan–Breslow–Wilcoxon test (χ^2^ = 21.48, df = 3, *p* < 0.0001). Median survival was 7.08 years for single-abutment TCDs and 10.33 years for TCDs supported by two to three abutments, whereas median survival was not reached for TCDs supported by four or more abutments. No prosthesis losses occurred in the subgroup supported by more than five abutments.

Among RCDs, prosthesis survival varied according to the number of abutments (Figure S3). The global log-rank test provided evidence of differences among the abutment-number groups (χ^2^ = 21.07, df = 2, *p* < 0.0001), with similar results obtained using the Gehan–Breslow–Wilcoxon test (χ^2^ = 15.83, df = 2, *p* = 0.0004). This subgroup analysis was exploratory. Median survival was 6.25 years for single-abutment RCDs, whereas it was not reached for RCDs supported by two or three abutments.

#### 3.5.2. Prosthesis Survival According to Denture Design

In a hypothesis-generating analysis, the interaction between prosthesis type and denture base design was examined in the restricted population (TCDs open: n = 289, 32 events; closed: n = 48, 11 events; RCDs open: n = 29, 12 events; closed: n = 219, 26 events).

Evidence of an interaction was observed (interaction HR = 0.18; 95% CI: 0.06–0.58), suggesting that the association between denture base design and prosthesis loss was different between RCDs and TCDs. Within the RCD group, closed base design was associated with a lower hazard of prosthesis loss than open design (HR = 0.32; 95% CI: 0.15–0.71), whereas the direction of the association was reversed among TCDs, with closed designs showing a higher—although not statistically significant—hazard of prosthesis loss compared with open designs (HR = 1.79; 95% CI: 0.80–3.72). Given the small size of some interaction cells and the clinically determined selection of denture design, these findings should be considered with caution.

To aid interpretation of these results, unadjusted Kaplan–Meier survival curves for the four groups defined by prosthesis type and denture design are shown in the Supplementary Materials (Figure S6).

## 4. Discussion

This single-center retrospective clinical cohort study analyzed the survival and clinical performance of telescopic-crown-retained dentures (TCDs) and root-cap-retained overdentures with ball attachments (RCDs). A total of 654 removable partial dentures, comprising 406 TCDs and 248 RCDs, were followed for up to 11.33 years. The influence of several patient- and prosthesis-related baseline parameters, including age, sex, jaw location, abutment number, Steffel support configuration, and denture design, on prosthesis survival, complication-free survival, and maintenance-free survival was analyzed.

Kaplan–Meier analysis of the complete cohort showed higher unadjusted prosthesis survival for TCDs than for RCDs, with estimated 5-year prosthesis survival rates of 90.6% and 80.2%, respectively, and prosthesis loss in 11.1% and 15.3% of restorations. In contrast, 5-year complication-free survival and complication rates were similar. Maintenance-free survival, however, differed markedly between groups. Median maintenance-free survival was markedly shorter for RCDs, with median times to first maintenance of approximately 35 days for TCDs and 6 days for RCDs.

The multivariable analyses provided a more differentiated interpretation of these unadjusted findings. After restricting the analysis to the overlapping range of one to three abutments and adjusting for abutment number, age, sex, and jaw location, prosthesis type was no longer independently associated with prosthesis loss (RCD vs. TCD: HR = 1.28; 95% CI: 0.75–2.19). Instead, single-abutment support was associated with a markedly increased loss hazard (HR = 3.67; 95% CI: 2.20–6.15), and point-like support was associated with prosthesis loss in the Steffel-based sensitivity analysis (HR = 2.88; 95% CI: 1.77–4.67). Thus, the lower unadjusted survival observed for RCDs appears to have been influenced substantially by their more compromised support situation rather than by the retention concept alone.

The adjusted analyses further showed that the three endpoints captured distinct dimensions of clinical performance. Evidence for a higher complication hazard with RCDs was model-dependent, whereas RCDs were consistently associated with earlier first documented maintenance across the adjusted analyses. Graphical diagnostics suggested that this difference was pronounced during early follow-up, while the interaction analysis indicated a greater difference among single-abutment restorations.

The 5-year prosthesis survival observed for TCDs in the present study is consistent with the generally favorable outcomes reported for removable partial dentures retained by telescopic or double crowns. Previous clinical studies have described telescopic-crown-retained dentures as reliable treatment options for partially dentate patients, although reported survival and success rates vary depending on study design, follow-up period, material, number and position of abutment teeth, and the definition of failure [7,18,30]. Wöstmann et al. [7] reported a 5-year survival probability of 95.1% for telescopic-crown-retained removable partial dentures and emphasized the influence of abutment number and structured aftercare on long-term success. Similarly, Schwindling et al. [18] reported an acceptable 7-year survival rate of 90.0% for double-crown-retained removable dental prostheses, while also demonstrating that minor technical complications and aftercare interventions were common. In a more recent practice-based cohort of non-precious alloy telescopic crowns, Wierichs et al. [30] reported favorable outcomes after up to 12 years and identified patient-, tooth-, and prosthesis-related factors associated with failure. Dentures supported by three or fewer telescopic crowns showed a higher risk of failure than dentures supported by more than three telescopic crowns.

These findings correspond closely with the present adjusted results, in which the support situation was more strongly associated with prosthesis loss than the retention concept itself. Single-abutment support emerged as the strongest prosthesis-related predictor of loss, while point-like support also showed an unfavorable association in the Steffel-based sensitivity analysis. From a biomechanical perspective, a higher number and more favorable distribution of abutments enlarge the support polygon, improve prosthesis stability, and distribute functional forces across a broader area. Conversely, single-abutment and point-like support permit greater rotational movement and concentrate loading on a limited supporting structure, which may explain their less favorable long-term prognosis.

The relevance of reduced support is not limited to TCDs. Root-cap-retained overdentures are commonly selected for patients with few remaining teeth, unfavorable abutment distribution, reduced coronal tooth structure, or compromised crown-to-root ratios [21,22,23,24,29,32,33]. These restorations may preserve residual roots, maintain periodontal proprioception, improve retention, and facilitate a later transition to complete edentulism if further tooth loss occurs [22,23,24,32,33]. The present findings do not question this therapeutic rationale. Rather, they indicate that prognosis in such compromised situations depends strongly on the number and distribution of the remaining abutments and should not be attributed to the retention concept in isolation.

The Steffel-based sensitivity analysis supported this interpretation. Point-like support was associated with prosthesis loss but not independently with complication-free or maintenance-free survival, suggesting that the prognostic relevance of support configuration depends on the endpoint considered. This endpoint-specific association is consistent with biomechanical concepts for double crown- and attachment-retained prostheses and current treatment-planning recommendations for severely reduced dentitions [9,26,34].

When the residual dentition provides an unfavorable support configuration, strategic implants may be considered to enlarge or improve the support polygon. Posterior implants beneath distal-extension removable partial dentures can convert resilient mucosal support into more stable implant-assisted support and may reduce rotational movement, loading of natural abutments, and patient discomfort [35]. Short strategic implants may be particularly relevant in elderly patients or in regions with limited vertical bone height because they can provide additional support while avoiding extensive augmentation procedures [34,36]. Such approaches should, however, be regarded as individualized treatment options and not as direct consequences of the present retrospective findings.

The findings for complication-free survival were less consistent across analytical approaches. The absolute complication rates and the unadjusted Kaplan–Meier analysis suggested broadly comparable complication patterns between TCDs and RCDs. In the primary adjusted model, however, RCDs showed a higher hazard of first complication. This association was attenuated in the Steffel-based sensitivity analysis, in which the confidence interval included unity. The evidence for an independent effect of the retention concept on complication-free survival should therefore be regarded as model-dependent rather than conclusive. Previous investigations have likewise shown that reported complication rates in removable prosthodontics vary according to the definition of the endpoint, follow-up duration, prosthesis design, and support characteristics [18,29,30,37]. Male sex and maxillary location were associated with lower complication hazards in the adjusted analyses; because these associations were not part of the primary clinical hypothesis and their underlying mechanisms cannot be determined from the retrospective data, they should be regarded as hypothesis-generating findings.

The maintenance results were more consistent and deserve particular attention. RCDs showed markedly shorter maintenance-free survival in the unadjusted analysis, and the primary Cox model yielded an average RCD-versus-TCD hazard ratio of 1.75 for first maintenance over the observed follow-up period. Graphical Schoenfeld-residual diagnostics suggested that this association was more pronounced during early follow-up and attenuated thereafter. The hazard ratio should therefore not be interpreted as representing a constant relative difference at every point in time. The average effect estimate remained nearly unchanged when Steffel support configuration replaced categorical abutment number. The very short median time to first maintenance, particularly in the RCD group, also reflects the inclusion of early post-insertion procedures such as pressure-point relief, occlusal adjustment, retention correction, and matrix activation as maintenance events.

The earlier first documented maintenance requirements observed for RCD are consistent with previous reports of frequent aftercare in root-cap-retained and tooth-supported overdentures. Stalder et al. [29] described frequent technical and biological complications in root-cap-retained overdentures, with matrix loosening representing a common technical event. Schuster et al. [33] reported that cover-denture prostheses supported by three or fewer abutments can remain functional over long observation periods but require repeated maintenance, including pressure-point relief, relining, management of decementation, and treatment of abutment-related complications. Chhabra et al. [38] likewise concluded that overdentures with metal copings may remain clinically serviceable while generating relevant post-procedural and maintenance needs. These findings support structured recall and realistic patient information regarding the expected frequency and nature of aftercare.

The interaction between prosthesis type and single-abutment support for maintenance-free survival adds further clinical differentiation. Among restorations supported by two to three abutments, RCDs already showed earlier first maintenance than TCDs, and the difference between the retention concepts was more pronounced among single-abutment restorations. This pattern may reflect the combination of limited biomechanical support and attachment-specific aftercare, particularly matrix activation or replacement. However, the association between abutment number and first maintenance was not monotonic: compared with two-abutment restorations, both one- and three-abutment restorations were associated with earlier first maintenance in the primary model, although the estimate for three versus two abutments was uncertain because its confidence interval was close to unity. Maintenance requirements may therefore reflect not only biomechanical support but also the number of retentive components, prosthesis complexity, and the clinical procedures needed to maintain adequate function.

The distinction between complications and maintenance is central to the interpretation of these findings. Studies of removable prostheses use heterogeneous definitions of success, survival, complications, and expected aftercare. Relining, loss of retention, pressure-point relief, or matrix adjustment may be classified as complications in some studies and as maintenance in others, which limits direct comparison. The present analysis separated complete prosthesis loss, first documented complication, and first maintenance intervention to provide a more differentiated assessment of clinical performance. This approach is consistent with systematic evidence emphasizing the broad spectrum of biological and technical events associated with removable dental prostheses and the importance of supportive care [37].

Biological maintenance remains relevant for root-retained overdentures. Moldovan et al. [37] emphasized that suitable pretreatment and supportive care may reduce biological complications, while Stalder et al. [29] linked such complications to plaque levels and denture-cleaning frequency. For RCDs, structured recall should therefore focus on repeated hygiene instruction and regular evaluation of the abutments, denture-bearing tissues, and retentive components.

Denture design represented an additional source of heterogeneity. An interaction between prosthesis type and open versus closed design was observed, suggesting that the association between prosthesis type and prosthesis loss differed according to denture design. Among open designs, RCDs showed a higher prosthesis-loss hazard than TCDs, whereas no clear difference between the retention concepts was observed among closed designs. Within the RCD group, closed designs were associated with a lower loss hazard than open designs, while no clear association with denture design was observed within the TCD group. However, these findings should be interpreted cautiously because some interaction cells contained few prostheses and events, and denture design was selected according to the underlying clinical and abutment situation. The observed associations may therefore be affected by sparse-data bias and confounding by indication and should be considered exploratory and hypothesis-generating.

The present design-related findings differ from those of Berger et al. [25], who reported lower abutment survival for closed root-retained overdentures and recommended an open design to facilitate hygiene access and saliva circulation. Open designs have also been discussed as advantageous for accessibility of the abutment region, whereas closed designs may facilitate later conversion into a complete denture [25,39,40,41]. However, the endpoints are not directly comparable: Berger et al. evaluated abutment survival, whereas the present analysis examined complete prosthesis loss.

Furthermore, the retrospective nature of the present study requires careful interpretation of the observed design effect. At our institution, root-cap-retained overdentures generally represent the final tooth-preserving treatment option for patients with severely compromised residual dentition. Consequently, the decision to fabricate an open or closed prosthetic design was not randomized but reflected the underlying clinical situation. Open-design RCDs were predominantly selected when the remaining abutments were considered sufficiently stable to allow a more hygiene-oriented design, whereas closed designs were more frequently chosen in cases with structurally compromised or strategically less favorable abutment teeth requiring additional denture support and stabilization. As a result, comparisons between open-design TCDs and RCDs are likely influenced by systematic differences in baseline abutment quality rather than by the denture design itself. By contrast, comparisons between closed-design TCDs and RCDs probably involve more similar clinical scenarios with respect to abutment prognosis. Therefore, the observed associations should primarily be interpreted as reflecting clinical treatment allocation rather than a causal effect of denture design.

Future prospective randomized studies should therefore evaluate telescopic-crown- and root-cap-retained overdentures with both open and closed designs in patients presenting with comparable baseline conditions, ideally restricted to one to three remaining abutment teeth. Such a study design would allow the independent effect of denture design to be distinguished from the influence of abutment quality and clinical case selection.

Based on the present data, no universal recommendation for an open or closed design can be made. Design selection should remain individualized and should consider abutment distribution, biomechanical stability, hygiene accessibility, future convertibility, anatomical limitations, and patient-related factors.

Clinically, RCDs should therefore not be regarded as an inherently inferior treatment concept. They are generally used in more compromised situations and may remain a pragmatic transitional or long-term option when the residual teeth are unsuitable for conventional clasp-retained or telescopic-crown-retained partial dentures and implant treatment is not feasible or desired. Their indication should be guided by the overall biomechanical situation and by patients’ hygiene capacity and ability to participate in structured supportive care, rather than by attachment type alone.

From a treatment-planning perspective, single-abutment and point-like support configurations appear relevant for prosthesis survival. If only one abutment remains, the indication should be evaluated carefully, and patients should be informed about the increased risk of prosthesis loss and, particularly for RCDs, early maintenance. Whenever clinically feasible, increasing the number of supporting units or improving their spatial distribution may enhance biomechanical stability; matrix-related maintenance should be incorporated into recall planning.

This tooth-preserving approach may be especially relevant in the mandible, where conventional complete denture rehabilitation is frequently challenging because of reduced stability, impaired masticatory function, and lower patient comfort [42,43,44]. Complete tooth loss can adversely affect oral comfort, function, psychosocial well-being, and nutritional status [42,43,44]. Comparable minimal-retention concepts, such as single-implant mandibular overdentures, have been discussed as simplified treatment options, especially for elderly patients, and have been associated with improved oral comfort and function [42]. As with RCDs, however, these reduced concepts may require frequent attachment-related maintenance, relining, or denture-base repair [29,42].

Several limitations must be considered. The retrospective design limited control over baseline variables, treatment allocation, recall intervals, and documentation quality. The decision to provide a TCD or RCD was based on the individual clinical situation, including the number, distribution, and strategic value of the remaining abutments, rather than on randomization. Accordingly, the groups differed substantially in age, jaw distribution, abutment number, Steffel support configuration, and denture design, and residual confounding by indication cannot be excluded. To improve comparability, the multivariable analyses were restricted to the overlapping range of one to three abutments. Although this reduced extrapolation across fundamentally different support situations, it excluded TCDs supported by four or more abutments and therefore limits the generalizability of the adjusted results to the full spectrum of telescopic restorations. Prosthesis type, abutment number, support configuration, and denture design were also closely interrelated, and their individual effects may not be completely separable.

These baseline imbalances may have influenced the observed outcomes through several pathways. Age may have reflected differences in general health, oral conditions, or treatment attendance, whereas jaw location, abutment number, support configuration, and denture design may have affected biomechanical loading, prosthetic support, and susceptibility to complications or prosthesis loss. Moreover, these variables were themselves related to the clinical indication for selecting a TCD or RCD and may therefore also represent markers of the underlying severity of the treatment situation. Although the adjusted analyses accounted for several measured differences, they cannot fully eliminate selection bias, residual confounding, or confounding by indication.

Treatment and follow-up were performed by different clinicians over the study period. Although all clinicians received the same initial instruction and followed standardized departmental treatment protocols, operator-related variability in clinical execution, assessment, and documentation may nevertheless have influenced the recorded outcomes. The prostheses were fabricated by two long-standing cooperating dental laboratories using coordinated laboratory protocols. Both laboratories performed calibration procedures and quality control before delivering the restorations; however, changes in individual technicians and potential material-related variation over the extended study period cannot be completely excluded. Conventional retentive and adhesive cementation protocols were applied according to standardized departmental procedures and remained consistent throughout the study period. Cementation mode was recorded separately but was not included in the present analysis because this investigation focused specifically on prosthesis-level outcomes. Accordingly, complications were restricted to documented prosthetic and technical events. Marginal gaps were additionally recorded because, in several cases, they were documented concurrently with prosthesis-related complications and could not always be reliably separated from the overall complication event in the retrospective records. Because the present investigation focused on prosthesis-level outcomes, the biological causes of abutment tooth loss were not systematically classified. Likewise, periodontal status, abutment mobility, crown-to-root ratio, endodontic status, remaining tooth structure, oral hygiene, recall adherence, smoking status, and systemic conditions were not available with sufficient completeness and standardization across the retrospective records to permit reliable inclusion in the analyses. Their potential influence on prosthesis survival and complication occurrence therefore could not be assessed, and residual patient- and abutment-level confounding cannot be excluded. Biological outcomes and the performance of the abutment teeth are being evaluated separately in an abutment-level analysis.

Patient-reported outcomes, including treatment satisfaction, oral health-related quality of life, and subjective functional perception, were not systematically documented and could therefore not be evaluated. Consequently, the present findings reflect prosthesis-level clinical outcomes but do not capture the patients’ subjective experience or perceived functional benefit. Future prospective studies should combine clinical survival and maintenance outcomes with validated patient-reported outcome measures.

The retrospective assessment depended on the completeness and consistency of the original clinical records. Although data extraction and event classification were cross-checked by two investigators and disagreements were resolved by consensus with a third experienced investigator, variability in the original detection and documentation of complications cannot be fully excluded. The maintenance endpoint combined early adaptation procedures with subsequent prosthetic maintenance and therefore represents the time to the first documented post-insertion intervention rather than cumulative long-term maintenance burden. Because the timing of early control visits and the clinical purpose of individual interventions were not standardized prospectively, early adaptation procedures and later maintenance could not be separated reliably. The timing of the first post-insertion visit may therefore have influenced ascertainment of the first maintenance event. Moreover, graphical Schoenfeld-residual diagnostics suggested that the RCD-versus-TCD association for first maintenance attenuated over time. The corresponding Cox hazard ratio therefore represents an average association over the observed follow-up period rather than a constant relative hazard.

The follow-up period was shorter in the RCD group, reducing the opportunity to observe late prosthesis losses, complications, and maintenance interventions. Although the Kaplan–Meier and Cox regression analyses accounted for unequal follow-up through right censoring, administrative censoring and censoring at the last documented follow-up were assumed to be non-informative; systematic differences in follow-up duration may nevertheless have introduced bias. Consequently, the cumulative burden of later events in the RCD group may have been underestimated, and estimates at longer follow-up times were based on fewer prostheses at risk. Complete prosthesis loss or remanufacture may preclude the subsequent occurrence of a first complication or maintenance intervention. Because prosthesis loss was censored at its occurrence, the Kaplan–Meier estimates should not be interpreted as cumulative incidence functions accounting for competing prosthesis loss, whereas the Cox regression models estimate cause-specific hazards. Death could not be considered as a competing event because reliable dates of death were unavailable. Minor events may also have been underreported if they were treated outside the department or not documented in detail. Some interaction and subgroup analyses included small numbers of restorations or few events, for example, three-abutment RCDs, triangular RCD configurations, quadrangular TCD configurations, and open RCDs. Consequently, these estimates may be statistically unstable, sensitive to individual events, and associated with limited precision and wide confidence intervals. Both the absence of statistically significant differences and isolated significant findings in these subgroups should therefore be interpreted cautiously. These analyses were secondary and exploratory and should not be regarded as providing definitive evidence. Furthermore, multiple exploratory comparisons were performed without formal adjustment for multiplicity, increasing the possibility of chance findings. The results should consequently be understood as clinically informative associations rather than definitive evidence of causality.

Despite these limitations, the study provides clinically relevant information by comparing two established retention concepts within one academic center and a standardized prosthodontic workflow. The cohort was substantial for a retrospective study involving root-cap-retained overdentures. Moreover, the separate evaluation of prosthesis survival, complication-free survival, and maintenance-free survival enabled clinically distinct outcome patterns to be identified. Restricting the adjusted analyses to the common abutment range, together with interaction and sensitivity analyses, provided a more differentiated assessment of the relationships among retention concept, abutment support, Steffel configuration, and denture design.

In summary, TCDs showed higher prosthesis survival than RCDs in the unadjusted complete-cohort analysis; however, retention concept was not independently associated with prosthesis loss after restriction to the common abutment range and multivariable adjustment. Single-abutment support, point-like support configuration, and increasing age were associated with prosthesis loss. Evidence for a higher complication hazard with RCDs was model-dependent, whereas RCDs were consistently associated with earlier first documented maintenance across the adjusted analyses. Graphical diagnostics suggested that this difference was particularly pronounced during early follow-up, while the interaction analysis indicated a greater difference among single-abutment restorations. The interaction with denture design further indicates that outcomes depend on the overall clinical, prosthetic, and biomechanical configuration rather than on the attachment system alone. RCDs therefore remain a relevant treatment option in severely reduced dentitions, provided that careful case selection, comprehensive patient information, and structured supportive care account for the expected maintenance requirements.

## 5. Conclusions

Within the limitations of this retrospective single-center study, telescopic-crown-retained dentures showed more favorable unadjusted prosthesis survival than root-cap-retained overdentures with ball attachments. After restriction to restorations supported by one to three abutments and adjustment for relevant covariates, however, the retention concept was not independently associated with prosthesis loss. Instead, single-abutment support and point-like support configurations were associated with an increased hazard of prosthesis loss, indicating that long-term prognosis is strongly influenced by the biomechanical support situation. Root-cap-retained overdentures showed earlier first documented maintenance, particularly during early follow-up and among single-abutment restorations, whereas evidence for an independently increased complication hazard was less robust across models. The observed interaction between retention concept and denture design further suggests that clinical outcomes depend on the overall prosthetic and biomechanical configuration rather than on the attachment system alone.

Nevertheless, root-cap-retained overdentures remain an important treatment option in severely reduced dentitions, as they may allow preservation of remaining roots and thereby avoid or delay complete edentulism. This may be relevant in the mandible, where conventional complete denture rehabilitation is often challenging. Despite their higher maintenance requirements, root-cap-retained overdentures may therefore represent a clinically valuable transitional or long-term treatment concept, provided that careful case selection, adequate patient information, and structured supportive care are ensured. Future prospective studies comparing root-cap-retained overdentures with conventional complete dentures are warranted, with particular emphasis on patient-reported outcomes such as oral health-related quality of life, chewing function, comfort, and patient satisfaction.

## Figures and Tables

**Table 1:** Demographic, prosthesis-related, and clinical characteristics of the study cohort. Data are presented as n (%) unless otherwise indicated. TCDs, telescopic-crown-retained dentures; RCDs, root-cap-retained overdentures; SD, standard deviation. Percentages were calculated within each prosthesis type.

Characteristic	TCD (n = 406)	RCD (n = 248)	Overall (n = 654)
**Age at prosthesis insertion, years**			
Mean ± SD	71.5 ± 11.8	75.4 ± 11.2	73.0 ± 11.7
Median (range)	72 (34–93)	75 (53–93)	73 (34–93)
**Sex**			
Female	195 (48.0%)	98 (39.5%)	293 (44.8%)
Male	211 (52.0%)	150 (60.5%)	361 (55.2%)
**Follow-up period, years**			
Mean (range)	4.15 (0.08–11.33)	2.75 (0.08–9.83)	3.61 (0.08–11.33)
Median	3.42	1.92	2.50
**Jaw**			
Maxilla	206 (50.7%)	66 (26.6%)	272 (41.6%)
Mandible	200 (49.3%)	182 (73.4%)	382 (58.4%)
**Prosthetic design**			
Open	355 (87.4%)	29 (11.7%)	384 (58.7%)
Closed	51 (12.6%)	219 (88.3%)	270 (41.3%)
**Steffel classification**			
Point-like	59 (14.5%)	123 (49.6%)	182 (27.8%)
Linear	292 (71.9%)	114 (46.0%)	406 (62.1%)
Triangular	47 (11.6%)	11 (4.4%)	58 (8.9%)
Quadrangular	8 (2.0%)	0 (0.0%)	8 (1.2%)
**Number of abutments**			
1	36 (8.9%)	113 (45.6%)	149 (22.8%)
2–3	301 (74.1%)	135 (54.4%)	436 (66.7%)
4–5	59 (14.5%)	0 (0.0%)	59 (9.0%)
>5	10 (2.5%)	0 (0.0%)	10 (1.5%)
**Clinical outcomes**			
Complete prosthesis loss	45 (11.1%)	38 (15.3%)	83 (12.7%)
At least one complication	78 (19.2%)	47 (19.0%)	125 (19.1%)
At least one maintenance intervention	314 (77.3%)	231 (93.1%)	545 (83.3%)

**Table 2:** Values are presented as n (%) of all prostheses in the respective treatment group (TCD, n = 406; RCD, n = 248). Only the first documented complication per prosthesis was considered. If two or more complication types were documented on the same earliest complication date, all simultaneous categories were counted. The categories are therefore not strictly mutually exclusive, and their sum may exceed the number of prostheses with at least one complication. Complications occurring after the earliest complication date were not included.

Complication Category	TCD (n = 406)	RCD (n = 248)
Marginal gap	4 (1.0%)	10 (4.0%)
Veneering repair	36 (8.9%)	10 (4.0%)
Acrylic resin repair	38 (9.4%)	21 (8.5%)
Framework repair	11 (2.7%)	10 (4.0%)
Hairline crack	10 (2.5%)	11 (4.4%)

**Table 3:** Values are presented as n (%) of all prostheses in the respective treatment group (TCD, n = 406; RCD, n = 248). Only the first documented maintenance event per prosthesis was considered. If two or more maintenance procedures were documented on the same earliest maintenance date, all simultaneous categories were counted. The categories are therefore not strictly mutually exclusive, and their sum may exceed the number of prostheses with at least one maintenance event. Maintenance procedures occurring after the earliest maintenance date were not included.

Maintenance Category	TCD (n = 406)	RCD (n = 248)
Pressure-point relief	218 (53.7%)	177 (71.4%)
Occlusal adjustment	123 (30.3%)	72 (29.0%)
Relining	130 (32.0%)	125 (50.4%)
Extension	82 (20.2%)	39 (15.7%)
Artificial denture tooth replacement	40 (9.9%)	5 (2.0%)
Excessive friction/retention	37 (9.1%)	not applicable
Insufficient friction/retention	23 (5.7%)	not applicable
Matrix adjustment/activation	not applicable	118 (47.6%)
Matrix replacement	not applicable	60 (24.2%)

**Table 4:** Adjusted multivariable Cox regression models for prosthesis loss. Data are presented as adjusted hazard ratios with 95% confidence intervals. Analysis was restricted to restorations supported by one to three abutments and adjusted for prosthesis type, sex, jaw location, age, and categorical abutment number. Reference categories were TCD, female sex, mandible, and two abutments. RCD, root-cap-retained overdenture; TCD, telescopic-crown-retained denture. The reported hazard ratios represent average associations over the observed follow-up period. Bold values indicate statistically significant associations (*p* < 0.05).

Covariate	Prosthesis Loss, Adjusted HR (95% CI) (n = 585; 81 Events)
RCD vs. TCD	1.28 (0.75–2.19)
Male vs. female	0.71 (0.45–1.13)
Maxilla vs. mandible	1.39 (0.87–2.21)
Age, per year	**1.05 (1.03–1.08)**
One vs. two abutments	**3.67 (2.20–6.15)**
Three vs. two abutments	0.52 (0.23–1.06)

**Table 5:** Exploratory multivariable Cox regression analyses. All analyses were restricted to restorations supported by one to three abutments (n = 585). Interaction models were adjusted for age, sex, and jaw location. Reference categories were TCD, female sex, mandible, 2–3 abutments. Main-effect estimates in interaction models are conditional on the stated reference category. Bold values indicate statistically significant associations (*p* < 0.05).

Outcome	Analysis	Comparison	Adjusted HR (95% CI)
Prosthesis loss	Exploratory abutment-interaction analysis	1 vs. 2–3 abutments within RCD	**4.69 (2.24–9.80)**
		1 vs. 2–3 abutments within TCD	**3.84 (1.83–7.51)**
		TCD vs. RCD within 1 abutment	0.65 (0.29–1.33)
		TCD vs. RCD within 2–3 abutments	0.82 (0.41–1.80)
		Interaction (prosthesis type × abutment support)	1.27 (0.48–3.66)

**Table 6:** Adjusted multivariable Cox regression models for first complication. Data are presented as adjusted hazard ratios with 95% confidence intervals. Analysis was restricted to restorations supported by one to three abutments and adjusted for prosthesis type, sex, jaw location, age, and categorical abutment number. Reference categories were TCD, female sex, mandible, and two abutments. RCD, root-cap-retained overdenture; TCD, telescopic-crown-retained denture. The reported hazard ratios represent average associations over the observed follow-up period. Bold values indicate statistically significant associations (*p* < 0.05).

Covariate	First Complication Adjusted HR (95% CI) (n = 585; 108 Events)
RCD vs. TCD	**1.81 (1.16–2.80)**
Male vs. female	**0.55 (0.38–0.82)**
Maxilla vs. mandible	**0.48 (0.31–0.73)**
Age, per year	1.00 (0.98–1.02)
One vs. two abutments	0.68 (0.40–1.13)
Three vs. two abutments	1.37 (0.85–2.16)

**Table 7:** Exploratory multivariable Cox regression analyses for complication-free survival. All analyses were restricted to restorations supported by one to three abutments (n = 585). Interaction models were adjusted for age, sex, and jaw location. Reference categories were TCD, female sex, mandible, 2–3 abutments. Main-effect estimates in interaction models are conditional on the stated reference category. Bold values indicate statistically significant associations (*p* < 0.05).

Outcome	Analysis	Comparison	Adjusted HR (95% CI)
First complication	Exploratory abutment- interaction analysis	1 vs. 2–3 abutments within RCD	0.67 (0.37–1.20)
		1 vs. 2–3 abutments within TCD	0.58 (0.20–1.32)
		RCD vs. TCD within 1 abutment	1.80 (0.66–5.07)
		RCD vs. TCD within 2–3 abutments	**1.62 (1.02–2.53)**
		interaction (prosthesis type × abutment support)	1.14 (0.40–3.79)

**Table 8:** Adjusted multivariable Cox regression models for first maintenance. Data are presented as adjusted hazard ratios with 95% confidence intervals. Analysis was restricted to restorations supported by one to three abutments and adjusted for prosthesis type, sex, jaw location, age, and categorical abutment number. Reference categories were TCD, female sex, mandible, and two abutments. RCD, root-cap-retained overdenture; TCD, telescopic-crown-retained denture. The reported hazard ratios represent average associations over the observed follow-up period. Bold values indicate statistically significant associations (*p* < 0.05).

Covariate	First Maintenance Adjusted HR (95% CI) (n = 585; 494 Events)
RCD vs. TCD	**1.75 (1.43–2.13)**
Male vs. female	0.99 (0.83–1.19)
Maxilla vs. mandible	0.90 (0.75–1.09)
Age, per year	**1.01 (1.01–1.02)**
One vs. two abutments	**1.52 (1.21–1.90)**
Three vs. two abutments	**1.25 (1.00–1.56)**

**Table 9:** Exploratory multivariable Cox regression analyses for maintenance-free survival. All analyses were restricted to restorations supported by one to three abutments (n = 585). Interaction models were adjusted for age, sex, and jaw location. Reference categories were TCD, female sex, mandible, 2–3 abutments. Main-effect estimates in interaction models are conditional on the stated reference category. Bold values indicate statistically significant associations (*p* < 0.05).

Outcome	Analysis	Comparison	Adjusted HR (95% CI)
First maintenance	Exploratory abutment- interaction analysis	1 vs. 2–3 abutments within RCD	**1.75 (1.34–2.30)**
		1 vs. 2–3 abutments within TCD	0.94 (0.62–1.37)
		RCD vs. TCD within 1 abutment	**2.65 (1.73–4.05)**
		RCD vs. TCD within 2–3 abutments	**1.45 (1.15–1.81)**
		Interaction (prosthesis type × abutment support)	**1.91 (1.20–3.11)**

## Data Availability

The original contributions presented in this study are included in the article. Further inquiries can be directed to the corresponding author.

## Supplementary Materials

The following supporting information can be downloaded at: https://www.mdpi.com/article/10.3390/dj14090545/s1, Figure S1: Kaplan–Meier estimates of prosthesis survival for TCDs and RCDs supported by one to three abutment teeth; Figure S2: Kaplan–Meier estimates of prosthesis survival for TCDs according to abutment number; Figure S3: Kaplan–Meier estimates of prosthesis survival for RCDs according to abutment number; Figure S4: Kaplan–Meier estimates of prosthesis survival according to prosthesis type and Steffel support configuration; Figure S5: Kaplan–Meier estimates of complication-free survival according to prosthesis type and Steffel support configuration; Figure S6: Kaplan–Meier estimates of prosthesis survival according to prosthesis type and denture design.
