# Supplementary material for: Prosthesis Survival, Complications, and Maintenance of Telescopic-Crown-Retained Removable Dental Prostheses and Root-Cap-Retained Overdentures: A Retrospective Cohort Study
Source: Dent J (Basel). 2026 Sep 1;14(9):545. doi: 10.3390/dj14090545 (PMC13605084; doi:10.3390/dj14090545)
Supplement: Supplementary file 1 [file dentistry-14-00545-s001.zip › dentistry-4472499-supplementary.pdf]

Article

Prosthesis Survival, Complications, and Maintenance of Telescopic Crown-Retained Removable Dental Prostheses and Root Cap-Retained Overdentures: A Retrospective Cohort Study

Anastasia Timoschenko [1,†], Lea Stoilov [1,†], Michael Marder [1], Helmut Stark [1], Norbert Enkling [1,2], Dominik Kraus [1,2,†] and Milan Stoilov [1,2,*,†]

1 Department of Prosthodontics, Preclinical Education and Dental Materials Science, University Hospital Bonn, 53111 Bonn, Germany; Anastasia.timoschenko@ukbonn.de; lea.stoilov@ukbonn.de; Michael.marder@ukbonn.de; helmut.stark@uni-bonn.de; enkling@uni-bonn.de; dominik.kraus@ukbonn.de; stoilov@uni-bonn.de

2 Department of Reconstructive Dentistry and Gerodontology, School of Dental Medicine, University of Bern, 3012 Bern, Switzerland; norbert.enkling@unibe.ch; dominik.kraus@ukbonn.de; stoilov@uni-bonn.de

† These authors contributed equally to this work

* Correspondence: stoilov@uni-bonn.de; M.S.

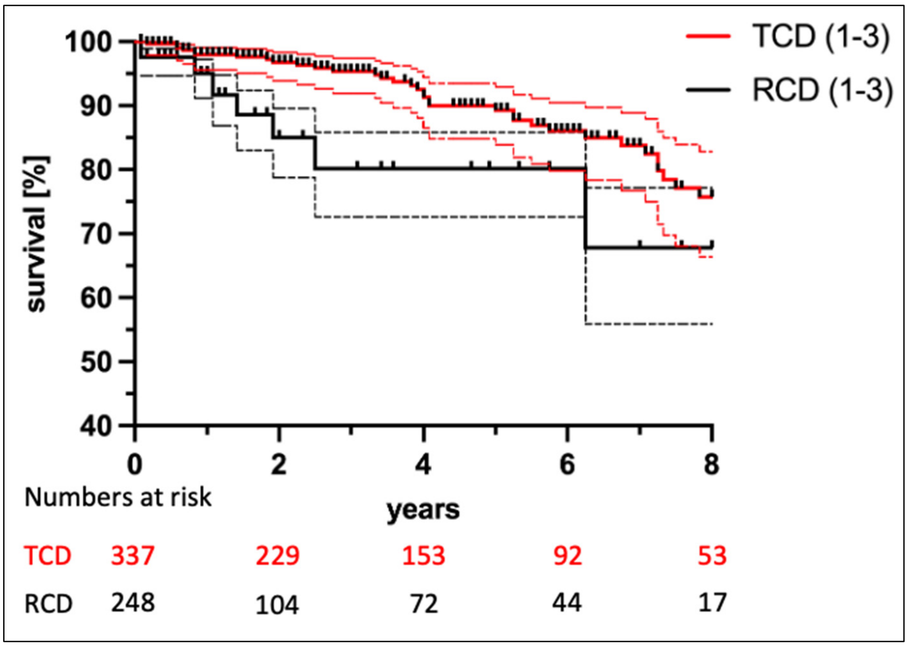

Figure S1: Kaplan–Meier estimates of prosthesis survival for telescopic crown-retained dentures (TCD) and root cap-retained overdentures (RCD) supported by one to three abutment teeth, with pointwise 95% confidence intervals calculated using the log–log transformation and numbers at risk. Prosthesis loss or remanufacturing was defined as the event. Curves are truncated at 8 years because few RCD remained at risk thereafter. Tick marks indicate censored observations. This unadjusted subgroup analysis was exploratory.

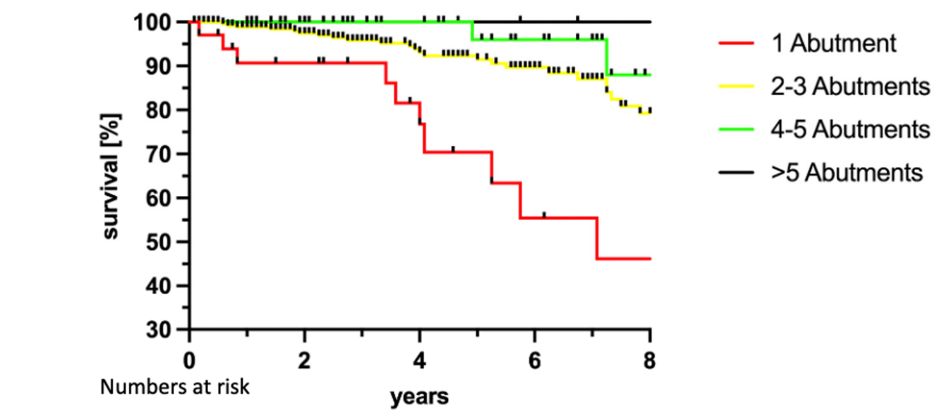

1 Abutment	35	27	17	8	6
2-3 Abutments	298	199	132	83	48
4-5 Abutments	59	46	32	21	8
>5 Abutments	8	7	6	4	3

Figure S2: Kaplan–Meier estimates of prosthesis survival for telescopic crown-retained dentures (TCD) stratified by the number of supporting abutment teeth, with numbers at risk. Prosthesis loss or remanufacturing was defined as the event. Curves are truncated at 8 years because only small numbers of restorations remained at risk in several abutment-number groups thereafter. Tick marks indicate censored observations.

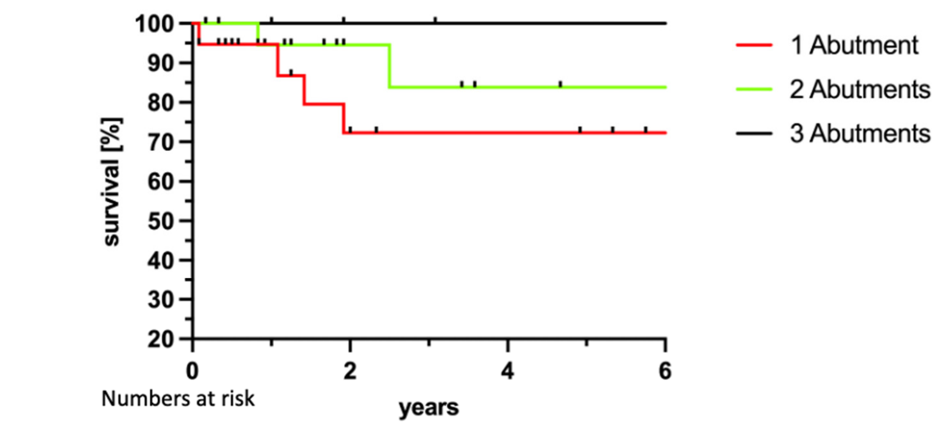

1 Abutment	113	50	38	16
2 Abutments	102	49	34	28
3 Abutments	33	22	10	10

Figure S3: Kaplan–Meier estimates of prosthesis survival for root cap-retained overdentures (RCD) stratified by the number of supporting abutment teeth, with numbers at risk. Prosthesis loss or remanufacturing was defined as the event. Curves are truncated at 6 years because only small numbers of restorations remained at risk in the abutment-number groups thereafter. Tick marks indicate censored observations.

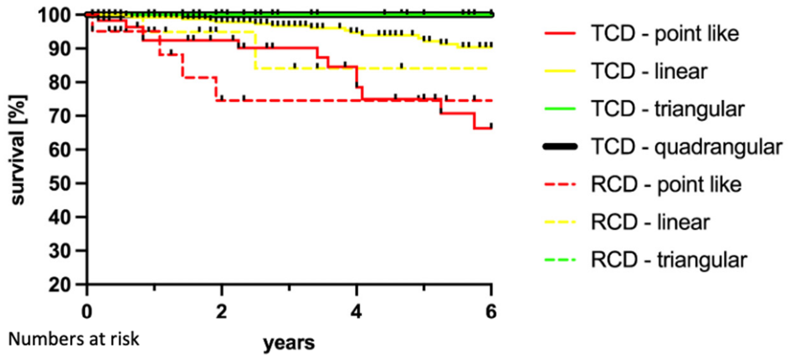

Numbers at risk

TCD - point like	59	43	28	15
TCD - linear	292	199	133	84
TCD - triangular	47	39	25	15
TCD - quadrangular	8	5	5	3
RCD - point like	123	55	43	21
RCD - linear	114	55	29	23
RCD - triangular	11	11	11	11

Figure S4: Kaplan–Meier estimates of prosthesis survival for telescopic crown-retained dentures (TCD) and root cap-retained overdentures (RCD) stratified by Steffel support configuration, with numbers at risk. The analysis included point-like, linear, triangular, and, where applicable, quadrangular support configurations. Prosthesis loss or remanufacturing was defined as the event. Curves are truncated at 6 years because only small numbers of restorations remained at risk in several support-configuration groups thereafter. Tick marks indicate censored observations. Some support-configuration groups were small from the outset and should therefore be interpreted cautiously.

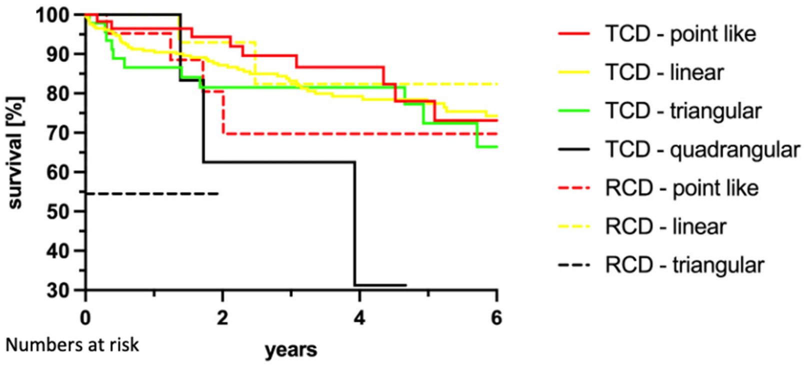

Numbers at risk

TCD - point like	59	41	27	14
TCD - linear	292	173	103	62
TCD - triangular	47	30	22	11
TCD - quadrangular	8	4	2	1
RCD - point like	123	50	39	21
RCD - linear	114	50	29	23
RCD - triangular	11	6	6	6

Figure S5: Kaplan–Meier estimates of complication-free survival for telescopic crown-retained dentures (TCD) and root cap-retained overdentures (RCD) stratified by Steffel support configuration, with numbers at risk. The analysis included point-like, linear, triangular, and, where applicable, quadrangular support configurations. The event was defined as the first documented prosthetic or technical complication. Curves are truncated at 6 years because only small numbers of restorations remained at risk in several support-configuration groups thereafter. Tick marks

indicating censored observations were omitted to maintain readability. Some support-configuration groups were small from the outset and should therefore be interpreted cautiously.

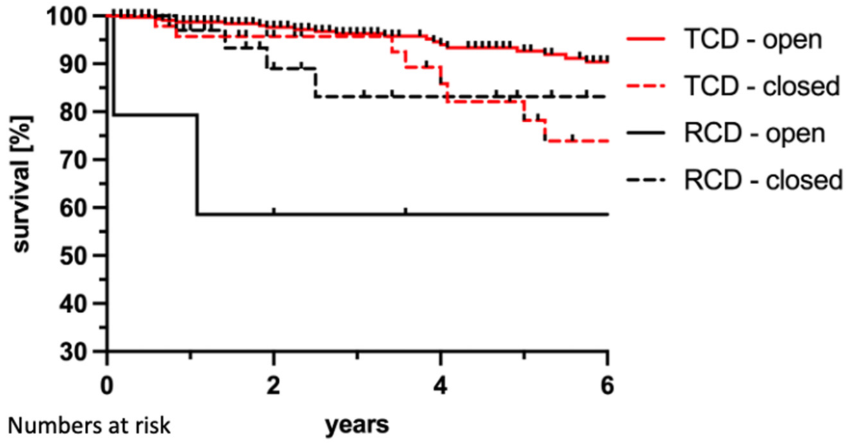

Numbers at risk

TCD - open	355	247	162	102
TCD - closed	51	35	26	16
RCD - open	29	17	11	11
RCD - closed	219	87	66	39

Figure S6: Kaplan–Meier estimates of prosthesis survival for telescopic crown-retained dentures (TCD) and root cap-retained overdentures (RCD) stratified by open versus closed denture design, with numbers at risk. Prosthesis loss or remanufacturing was defined as the event. Curves are truncated at 6 years because only small numbers of restorations remained at risk in several prosthesis type–design groups thereafter. Tick marks indicate censored observations. Some groups were small from the outset, and the findings should therefore be interpreted as exploratory.
